# Correction

**DOI:** 10.1111/cas.12403

**Published:** 2014-04-11

The authors would like to draw the reader's attention to an error in the following article:

Yuichi Ando, Megumi Inada-Inoue, Ayako Mitsuma, Takayuki Yoshino, Atsushi Ohtsu, Naoko Suenaga, Masahiko Sato, Tomoyuki Kakizume, Matthew Robson, Cornelia Quadt and Toshihiko Doi

Phase I dose-escalation study of buparlisib (BKM120), an oral pan-class I PI3K inhibitor, in Japanese patients with advanced solid tumors

*Cancer Sci* 2014; **105**: 347–353. This article has been published on Early View on February 13, 2014.

On page 352, one of the doctors’ names in the Acknowledgement section has been incorrectly presented.

“Dr Akifumi Kato” should be corrected to:

“Dr Terufumi Kato”

The authors apologize for this error and any confusion it may have caused.

